# Overcoming therapeutic MAb resistance in agressive HER2 positive breast carcinomas by adoptive immunotherapy using optizimed effectors cells

**DOI:** 10.1186/2051-1426-1-S1-P176

**Published:** 2013-11-07

**Authors:** Sandrine Valsesia-Wittmann, Beatrice Clemenceau, Anne-Catherine Jallas, Jean-Yves Blay, Aurelien Marabelle, Christophe Caux, Henri Vie

**Affiliations:** 1 P3I, Centre Leon Berard, Lyon, France; 2 INSERM CRCNA U892, Universite de Nantes, Nantes, France

## 

Herceptin (Trastuzumab), a monoclonal antibody targeting HER2 has been demonstrated to improve survival of HER2 overexpressing metastatic breast cancer. However, half of patients who initially respond to Herceptin develop resistance within one year of treatment initiation, and in the adjuvant setting 15% of patients still relapse after one year of treatment despite Herceptin-based therapy. Alteration of Antibodies Dependant Cellular Cytotoxicity (ADCC) is one of the rational mechanisms for resistance to therapeutic Mab. The goal of this project is to overcome this resistance by adoptive transfer using genetically engineered optimized NK cells. We thus compare efficacy of two different approaches : (i) NK cells armed with a high affinity Fc domain (FcγRIIIα, CD16) linked to its transduction chain FCεRIγ (CD16/γ) to allow recognition and interaction with Mab, inducing ADCC and (ii) NK cells expressing Herceptin sequence fused to the transduction chain FCεRIγ(CD16/γ) to directly kill the HER2 positively cells. Results obtained in vitro demonstrated higher direct killing efficacy of NK expressing Herceptin/γ against HER2 amplified breast carcinoma cells. However, abnormal reactivity against cells with undetectable HER2 expression by FACS analysis was observed. This last point might represent an important limitation point for clinical use. On the other hand, “two step killing by ADCC” with NK-CD16/γ demonstrated a very specific cytotoxicity against HER2 positive cells through ADCC only in the presence of antibody. We demonstrated that efficacy of specific lysis is linked (i) to effector dose, (ii) levels of CD16/γ expression on effectors cells, (iii) HER2 expression but not level on target cells In Herceptin resistant HER2+ xenograft immunodeficient NSG mice model, complete regression was obtained only with NK- CD16/γ in the presence of Herceptin. Our last results will be discussed. Restoration or improvement of effectors cells might be considerate as an issue in therapeutic humanized Mab resistance treatment. Successful immune-based therapies will likely ultimately integrate strategies that combine immunotherapy approaches and immune-modulating drugs, in order to maximize their antitumor activity.

